# A Survey of Insulin-Dependent Diabetes—Part I: Therapies and Devices

**DOI:** 10.1155/2008/639019

**Published:** 2008-01-27

**Authors:** Daisuke Takahashi, Yang Xiao, Fei Hu, Michael Lewis

**Affiliations:** ^1^Department of Computer Science, The University of Alabama, Tuscaloosa, AL 35487-0290, USA; ^2^Department of Computer Engineering, Rochester Institute of Technology, Rochester, NY 14623-5603, USA

## Abstract

This paper surveys diabetes therapies from telemedicine viewpoint. In type 1 diabetes therapies, the exogenous insulin replacement is generally considered as a primary treatment. However, the complete replacement of exogenous insulin is still a challenging issue because of its complexity of modeling the dynamics, which is typically modeled nonlinearly. On the other hand, thanks to the progress of medical devices, currently the diabetes therapies are being automated. These medical devices include automated insulin pumps and blood glucose sensors. Insulin pumps are designed to create artificial insulin perfusion while they largely rely on the blood glucose profile measurements and these measurements are achieved by one or more blood glucose sensors. The blood glucose measurements are also important for the insulin-dependent diabetes therapies. An insulin pump along with sensors establishes a good feedback system providing the appropriate amount of the exogenous insulin on demand. Controlling the amount of exogenous insulin to suppress the blood glucose levels requires complicated computations. This paper mostly explains both type 1 and 2 diabetes and their mechanisms accompanied by descriptions of diabetes therapy and medical devices currently utilized in the therapy.

## 1. INTRODUCTION

Diabetes mellitus is a metabolic disorder resulting from the permanent lack of insulin production from the pancreas (type 1 diabetes) or the chronic degradation of the functionality of endogenous insulin (type 2 diabetes), which results in raising the glucose concentration in blood because without insulin, the cellular system cannot properly convert carbohydrates such as sugars, starches, or other foods into energy usable by the body. These factors eventually result in several complications, such as cardiovascular disease, chronic renal failure, retinal damage, nerve damage, and microvascular damage [1]. Both types 1 and 2 diabetes are chronic and currently incurable, seemingly linked to genetics.

In addition, diabetes is a condition that disproportionately affects developed countries. From a report of the World Health Organization (WHO), currently, around 180 million people suffer from diabetes all over the world, and it is thought that over 350 million people will suffer from diabetes by the year 2030. Besides, the number of people died from diabetes was approximately 1.1 million in 2005, and half of this number is aged under 70 years old [2]. It is also thought that 136 billion dollars are spent annually in the United States for 12 million diabetes patients [3, 4].

Current treatment for diabetes can include home-administered care under the guidance of a physician. Intensive treatment can mitigate the effects of existing conditions as well as reducing the risk of developing advanced complications, such as those previously listed.

Early methods of home care involved using logs and tables, applying diet and exercise to predetermined doses prescribed by the patient’s doctor. Modern microcontrollers, sensors, and pumps now allow for the automated administration of insulin within doctor-prescribed parameters. Further, technological advances currently permit these devices to be wearable, acting as an *artificial pancreas*. The goals of such a product include being safe, automatic, and nonintrusive. These devices must employ an effective control scheme that allows the blood glucose (BG) level to be kept within a safe range of nominal.

This paper overviews diabetes therapies and devices from a telemedicine viewpoint. We mostly discuss the insulin-dependent diabetes therapy or type 1 diabetic therapy although we may occasionally mention the noninsulin-dependent one. It first briefs both types of diabetes mellitus with respect to the mechanisms of endogenous insulin deficiency in Section 2. In Section 3, we outline a basic procedure of the therapy and address the difficulties and the goals. In Section 4, we survey several ways, in which the exogenous insulin is injected, and their effect. In Section 5, we present some commercially available medical devices particularly for diabetes as well as those in research. Then Section 6 is spent to briefly explain control theories used for the diabetes therapy. Due to the limit space and complexity of controlling the amount of exogenous insulin to suppress the blood glucose levels, control methods are briefly discussed. Details about the control schemes are included in another paper [5]. Finally, we conclude our paper in Section 7.

## 2. DIABETES MELLITUS

Diabetes has been recognized since about 2000 B.C. However, until the discovery of insulin in 1921, diabetes was properly treated so that the insulin-dependent diabetic could delay the emergence of the complications [6, 7]. A characteristic of diabetes mellitus is the high blood glucose concentration mainly caused by the lack of internal insulin production, which is the principal hormone and suppresses the rising of the blood glucose concentration by turning it into a main energy required by the human body [1]. While blood glucose is used as energy to work muscles, the brain, and all other tissues, excessive concentration of glucose in blood leads to several complications. Cardiovascular disease, chronic renal failure, retinal damage, nerve damage, and microvascular damage are known as serious complications caused by diabetes.

According to mechanisms in which insulin deficiency occurs, the World Health Organization categorizes two types of diabetes mellitus: type 1 and type 2 diabetes [1]. The characteristics of type 1 diabetes are the permanent lack of insulin production from the pancreas due to the destruction of the *β* cells of the Islets of Langerhans. In this kind of diabetes, insulin replacement is required to compensate its production by the therapy.

On the other hand, in type 2 diabetes, the functionality of internal insulin becomes gradually degraded, and metabolisms that exchange blood glucose into a main energy work less than that in the normal person. This condition keeps blood glucose unused and eventually leads to hyperglycemia, which may cause eye, kidney, and nerve damage [8]. In the early stage, exercises and controlled regimens can improve, but not cure, this metabolic disorder. However, leaving type 2 diabetes untreated, hyperglycemia requires exogenous insulin inputs like the type 1 diabetic therapy.

### 2.1. Blood glucose and insulin

From its metabolism, blood glucose or blood sugar derived from a form of carbohydrate eventually turns into energy required by the human body and also works the brain and the nervous system. Glucose is typically carried throughout the human body via blood vessels as a form of red blood cell and plasma. In the human body, only insulin and glucagon play a role of regulating blood glucose levels within a very narrow range by bearing it from blood to the most cells, such as muscles and adipose tissues, turning it to energy [8, 9].

Typically, either excess or shortage of glucose in blood is known as a metabolic disorder. The condition where the blood glucose is much lower than expected is called hypoglycemia causing drowsiness, mental malfunctioning, irritability, and loss of consciousness [8]. To the contrary, the condition where the blood glucose levels are much higher is called hyperglycemia, and long-term hyperglycemic conditions eventually result in diabetes [8].

As we already mentioned, insulin strictly regulates blood glucose levels. Insulin is a polypeptide hormone and a production of the *β* cells of the Islets of Langerhans in the pancreas. The main activities of endogenous insulin are mainly two things: to help the liver yield glycogen to be preserved from glucose, and to help muscles and adipose tissues intake glucose to be broken down into energy. However, the lack of insulin in the liver forces glycogen to turn back to glucose that is ejected into blood vessels. Also, the condition leads to keep glucose in blood not processed into energy. Thus the lack or the shortage of the insulin production by the pancreas immediately causes metabolic disorders, that is, hyperglycemia or high blood glucose concentration, thus eventually resulting in diabetes [9].

### 2.2. Type 1 diabetes

In type 1 diabetes, the *β* cells in the pancreas are destroyed by the immune system which should respond to an infection caused by viruses, such as the Coxsackie virus family or German measles [10]. As a result, the pancreas stops supplying insulin into blood causing high blood glucose concentration. Although type 1 diabetes is occasionally called childhood, juvenile, or insulin-dependent diabetes, it is not a metabolic disorder that emerges only during a childhood but after growing up to adults [10].

When type 1 diabetes is untreated for a long time, it eventually results in diabetic coma, diabetic ketoacidosis, or death [10]. Diabetic ketoacidosis comes from a fact that when blood glucose cannot be used in a form of energy for the body due to the lack of insulin, instead, proteins or fats are used to be turned into a main energy. This metabolic process involves several chemical reactions, such as oxidization of the fatty acid resulting into *acetyl coenzyme A* (CoA), and a sequence of the citric acid cycle and the respiratory chain where acetyl coenzyme is eventually turned into energy by an electron transport chain [11–14]. However, the process of these chemical reactions also creates ketones as by-products, and because of their acidity, ketones turn blood into acid resulting in acidosis or ketoacidosis.

Treatment of type 1 diabetes is to compensate for the loss of endogenous insulin usually by infusing exogenous insulin into the body. This artificial insulin supplement suppresses a rise of blood glucose concentration and delivers blood glucose to the most cells through the body. The insulin injections are conducted three or four times per day, or alternatively, an insulin pump is utilized to continuously deliver a little fast-acting insulin for the basal insulin supply [15]. These treatments of type 1 diabetes are known as the insulin-dependent diabetes therapy.

### 2.3. Type 2 diabetes

Type 2 diabetes emerges in conditions of insulin resistance, insulin deficiency, and hyperglycemia [16]. In insulin resistance, since functions of internal insulin are largely degraded, it cannot appropriately facilitate the insulin-glucose metabolism, so regardless of its existence, body cells cannot take in blood glucose enough for energy supply [17]. Eventually, type 2 diabetes stimulates hyperglycemia, which is a condition where blood glucose levels keep much higher than expected for a long time, it may cause eye, kidney, and nerve damage [8]. However, in its initial stages, the symptoms of the disease are not so serious that they cannot be often realized until they are brought up to a more critical and chronic situation [17]. Type 2 diabetes is mainly caused by several irregular lifestyles, such as the lack of exercise, obesity, or a sedentary lifestyle [16]. Meanwhile, genetic factors also cause this type of diabetes.

Type 2 diabetes is also called noninsulin-dependent, obesity related, or adult-onset diabetes.

Treatment in the earlier phases of type 2 diabetes is comparatively easier than type 1 diabetes because of the existence of more or less internal insulin production [17]. The treatment in the initial stages mainly consists of glycemic control, which keeps blood glucose levels normal to prevent hyperglycemic stimuli [18], and lifestyle control, which provides patients with desired meals and exercises from the aspect of the personal nutrition, and typically supported by a variety of medical staffs [17]. However, as type 2 diabetes grows worse, the antidiabetic drug therapy is necessary in addition to glycemic and lifestyle controls. Moreover, if these therapies cannot help managing blood glucose levels, further treatments, such as insulin therapy, are required besides the oral medication therapy.

The reason or origin of type 2 diabetes is still unknown in etiology, and currently, no one can be cured completely from type 2 diabetes [17].

## 3. INSULIN-DEPENDENT DIABETES THERAPY

**Figure 1:**
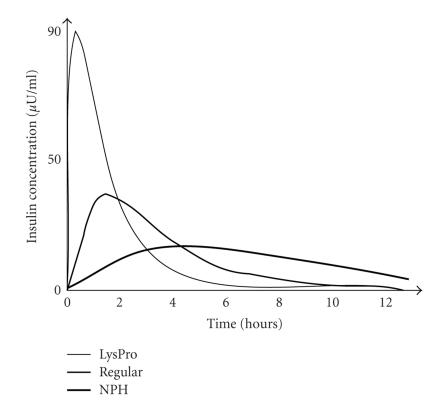
An example of insulin pump that integrates a blood glucose sensor [21]. Patient’s blood is sampled from the tip of his/her finger, and from the direct measurements of a blood glucose sensor, the amount of the short-acting insulin is adjusted and continuously delivered into the human body.

A basic idea of insulin-dependent diabetes therapy is initiated by a report of the Diabetes Control and Complication Trial, that is, the well-controlled glucose metabolism manages well or delays the emergence of the complications of insulin-dependent diabetes [15, 19, 20]. Thus in typical cases of the insulin therapy, according to blood tests, three or four times of insulin injections are subcutaneously carried out per day to compensate the lack of endogenous insulin secretion for type 1 diabetes in order to suppress the rise of blood glucose levels [10]. Sample blood is typically picked from the tip of a finger or earlobe, and the amount of blood glucose is checked using a sensor [15]. For example, thanks to the development of the NPH (neutral protamine hagedorn) insulin, the combination of the NPH and regular insulin is injected before breakfast. Usually, the effect of the NPH insulin is very slow and continues for a long period, which resembles the basal supply of endogenous insulin for a normal person. Meanwhile, the regular insulin makes up for the bolus supply of endogenous insulin during a meal intake, so the regular insulin acts immediately and whose peak is higher than the NPH insulin, shown in Figure 1. Then before dinner and bedtime, a couple of other regular insulin injections are carried out according to test results [6].

Although the subcutaneous insulin infusion is expected to follow precisely the complex real hormone secretion, the conventional insulin therapy does not follow this complex system enough neither temporally nor quantitatively yet. In fact, the metabolism of a nondiabetes person controls blood glucose levels in a quite narrow range all the time [6], for example, within the range of 4 to 8 mmol/L [22].

Thus one goal of the insulin-dependent diabetes therapy is to precisely capture the insulin-glucose dynamics of the normal person and design a system yielding the insulin secretion quantitatively as close to the real metabolism as possible. However, in practice, many parameters around the metabolism, such as environmental conditions, stress of patients, behavioral changes, and duration of tests make a calculation of the actual demand of exogenous insulin intricate [23]. As a result, designed algorithms and mathematical representations must be complex and nonlinearly modeled in order to resemble the real hormone secretion [24].

Meanwhile, to support the therapy, short-term in vivo glucose sensors have been recently integrated into insulin-dependent diabetes therapeutic system [25]. The in vivo glucose sensors typically are implanted subcutaneously and send blood glucose profiles as vital signals to exterior receivers, such as PDAs or laptop PCs, at very short intervals. Since many samples make modeling a blood glucose curve relatively easy resulting in enhancing predictions of the future transition of blood glucose concentration [24], the in vivo sensors are considered to be largely beneficial to a reliable insulin-dependent diabetes therapy. Currently in collaboration with an insulin pump, in vivo blood glucose sensors are integrated into closed-loop blood glucose control systems.

Problems of current in vivo blood glucose sensors are, however, that reliable long-term blood glucose monitoring is still challenging and is not approved by U.S. Food and Drug Administration (FDA). Currently, only three-day monitoring is considered to be safe and reliable [24, 26]. In addition, these blood glucose sensors largely still rely on the discrete measurements rather than continuous ones [24].

Broadly speaking, as types of the insulin therapy, three models are mainly utilized, for example, open-loop, closed-loop, and partially closed-loop control models. These models have their own advantages and disadvantages for their use. Among the three models, the open-loop control and the partial closed-loop control require physician’s assistance for injecting the exogenous insulin, whereas in the closed-loop control according to feedbacks from the output of blood glucose sensors, the amount of the exogenous insulin is determined so that the system is ideally autonomous and self-organized. Examples of the closed-loop control schemes are pole-assignment strategy and self-tuning adaptive control, and examples of the partially closed-loop controls are the automated insulin dosage advisor (AIDA) and the diabetes advisory system (DIAS) [15].

## 4. METHODS OF INSULIN INJECTION AND INFUSION

There are several ways to inject insulin into the human body, that is, intravenous, subcutaneous, and intraperitoneal routes. The most direct method is intravenous infusion [15], while subtler ways can be used for continuous or less painful dispensation, such as subcutaneous injection. Furthermore, the method of injection as well as the control method places a number of requirements on the type of insulin applied to the patient. In either way of the insulin infusion, the objective is to keep the condition of normoglycemia for the type 1 diabetic like normal people at all times.

### 4.1. Intravenous insulin injection

One way for the exogenous insulin injection is to utilize an intravenous route. Intravenous infusion works by injecting drugs directly into a patient’s blood stream through a needle, which penetrates the skin, and into a vein. Several advantages of the intravenous insulin injection benefit for insulin-dependent diabetes therapy. Compared with the other routes, to inject insulin intravenously delivers insulin more quickly and helps it reach the bloodstream in the higher ratio. In addition, against the overdose of insulin, utilizing this route can enhance the sensitivity to hypoglycemia, respond immediately and stop delivering. Furthermore, this route of insulin injection is considered to be better suited for improved closed-loop mechanisms [27].

However, despite of a lot of benefits, the intravenous route also includes a couple of shortcomings. For example, in the intravenous insulin infusion, since the catheter is kept to be put into the vein, this may cause the blood vessel to be irritated. Also, in a long time insulin infusion, the catheter may be put away from the vein or it may be obstructed [27].

One experiment showed that for the type 1 diabetic, a continuous crystalline insulin infusion intravenously could improve the insulin profile prominently close to the normal for 30 minutes from the beginning of a meal [6]. To the contrary, the same experiment reported the anomaly of the glycemic profile where the blood glucose concentration regained above normal after the initial 15–30 minutes of the experiment [6].

Moreover, to achieve the normalization of the glycemic and metabolic profile with an ambulatory insulin-dependent diabetes therapy, delivering insulin in the central venous route with a continuous open-loop infusion system is feasible [28].

### 4.2. Subcutaneous insulin injection

An alternative way to inject exogenous insulin is the subcutaneous route that is much simpler and less risky of infection at an injection site than in an intravenous route, although it presents certain design challenges for blood glucose level control. It can be used to facilitate continuous, noninterrupted glucose management. Due to its relatively pain-free application (compared to intravenous infusion), it is much more agreeable to home monitoring patients who wear artificial pancreas devices at all times [29]. Therefore, basically insulin injections and infusions are conducted safely by the diabetic on their own. Most of the clinical experiments demonstrated that, compared with conventional single injections, to infuse insulin subcutaneously administrates the glycemic profile better. Also, it was turned out that by delivering insulin mechanically, long-term subcutaneous experiments regulated values of hemoglobin A_1_C for the diabetic [6].

However, occasionally infusing insulin subcutaneously is not sufficient for reversing diabetic complications, particularly brittle patients, because of its limitations of controlling the glycemic profile [6].

### 4.3. Intraperitoneal insulin injection

Although only a limited number of experiments were carried out, insulin can be injected in an intraperoneal route. Early experiments of insulin-dependent diabetes therapy with the intraperitoneal insulin infusion showed that insulin soaked up from the peritoneum would appear in the surrounding blood system of the diabetic with peritoneal dialysis. Meanwhile, it was possible for diabetics to have daily activities and ambulate as usual. In addition to these benefits, the same experiments demonstrated no diabetic complications by daily basis catheters replacement [6, 30].

Furthermore, in the intraperitoneal insulin infusion, insulin is soaked up into the blood system moderately in the range of subcutaneous insulin injection to intravenous insulin infusion, while the insulin is delivered from the peritoneum directly to the liver resulting in the reduction of high concentration of peripheral insulin [6].

On the other hand, despite of its advantages, in the intraperitoneal insulin infusion, external insulin delivery systems increase the risk of infection developing peritonitis as well as tissue degradation at the catheter site [6].

### 4.4. Comparison of methods

Because it is less invasive to the body, subcutaneous injection is generally safer for several reasons [15]. Persons with an active lifestyle are put at risk by leaving an embedded needle protruding from their body, necessary to bridge the fluids gap between their bodies and their artificial pancreas. Subcutaneous administration greatly reduces this safety hazard by interfacing no deeper than the skin layers, often distributed over an area, decreasing the invasiveness of the device [29]. Furthermore, continuous needle-sticking as well as prolonged embedding poses health risks of infection, clotting, or other sorts of body-triggered rejection to an invasive foreign device. Because subcutaneous injection is far less invasive, it is less likely to trigger self-defense mechanisms like this, and if triggered, they tend to be far less severe.

However, subcutaneous injection is not without its drawbacks. Due to its less invasive nature, insulin takes far longer to permeate into the body than through direct intravenous infusion. It has been reported that when using standard insulin for both injection types, subcutaneous injection can take as much as three times longer to take effect. This makes implementing an accurate control system quite challenging due to the time delays.

### 4.5. Insulin selection

Several options are available for insulin to be injected, depending upon the situation [15]. Regular insulin has historically been used to treat diabetes. It can be both intravenous and subcutaneous injected, depending upon the injection interface used. When it is subcutaneously injected, its effects take up to three times longer than if it is intravenously injected, posing a difficult control systems problem.

An insulin preparation by the name of Lispro was developed to aid in the rapid absorption of the insulin [15]. It is designed to take immediate effect, and can be fully absorbed into the body’s system significantly faster than regular insulin. Lispro is designed to take effect within 15 minutes and peak about an hour after application. Because of its fast-delivery capabilities, Lispro is the most commonly used with subcutaneous injection. Subcutaneous injected Lispro has been proven to take effect within approximately the same time frame as intravenous injected normal insulin. This enables home monitoring and injection patients to control their glucose levels to the same levels as with intravenous management, but in a far less invasive and painful way.

An insulin preparation by the name of NPH has also been developed for the exact opposite purpose of Lispro [15]. NPH is designed to take effect over a much longer period of time, lasting up to 24 hours. The purpose of insulin with this property is to provide the patient with a predictable baseline level of insulin throughout the day, allowing the control system to focus its efforts on accounting for and correcting anomalies and disruptions that are encountered, such as meals, exercise, and sleep.

Appropriate injections of a combination of Lispro and NPH has been proven incredibly effective in maintaining short and long-term glucose stability within patients using a continuous monitoring and control device [29]. Lispro is usually used for fast-acting control to counterbalance blood glucose fluctuations due to meals. NPH is usually used to provide a constant trickle of insulin in order to establish a baseline, known as a *basal* level.

## 5. DEVICES IN INSULIN-DEPENDENT DIABETES THERAPY

### 5.1. Insulin pump

**Figure 2:**
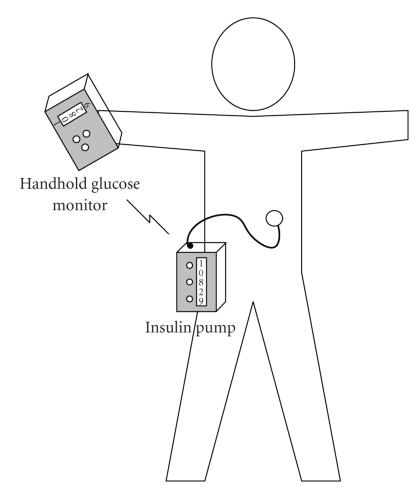
An image of an insulin pump. This image also includes a blood glucose sensor. In the general use, an insulin pump continuously infuses a little amount of the short-acting exogenous insulin via an attached needle as a basal dosage. It is also able to add extra exogenous insulin for intensive care, for example, meal time. Insulin pumps have a lot of advantages compared with the insulin shots.

**Figure 3:**
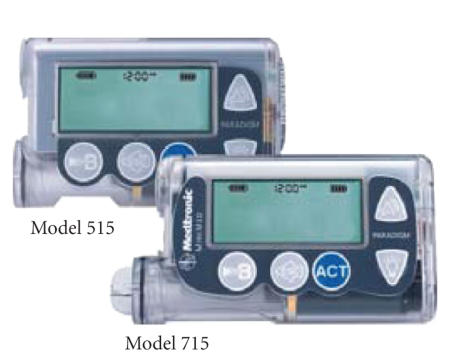
Another example of insulin pumps of Medtronic. A processing module has a small display that shows a current rate of an exogenous insulin infusion. It also has several control buttons just below the display. Two of these buttons are used to manage a current insulin infusion rate.

Insulin pumps are used to create an artificial insulin secretion subcutaneously that can suppress excessive blood glucose propagation for the type 1 diabetic. In general, it continuously delivers a basal rate of exogenous insulin automatically and also has capability of manually adding extra insulin to the basal rate for the intensive insulin secretion by a continuous subcutaneous insulin infusion (CSII) technique [31]. Most of the insulin pumps consist of a small processing module with a display, a disposable insulin reservoir, and an insulin syringe, and are powered by batteries, illustrated in Figures 2 and 3. The amount of insulin supply is externally controllable depending on blood glucose measurements.

In the use of an insulin pump, after finishing setting-up, a patient inserts a cannula into his/her abdomen as seen in Figure 2. Upon determining an insulin infusion rate, it starts delivering exogenous insulin continuously in 24 hours per day and 7 days a week, that is, a basal dose [32]. During meal time, when more amount of the insulin dosage is required because of carbohydrate intakes, the insulin pump enhances its insulin supply to adjust high blood glucose concentrations, which is called a bolus dose so that this artificial insulin metabolism should occur after a real physiological metabolism in the normal human body.

In case of the insulin injection using a syringe, since the insulin dosage is performed three or four times a day, each shot is devised in such a way that the insulin dosage can keep its effect constantly as long as possible. In general, using a delayed action insulin or slow-acting insulin helps a basal supply of exogenous insulin. A difficulty of the insulin injection, however, results from using this slow-acting insulin. Although the duration of the effect is achieved longer than other types of insulin, it also takes a longer time to start taking effect as illustrated in Figure 1. This disadvantage largely prevents effective predictions of a future blood glucose profile. In this connection, its variance eventually grows up to 52% [31]. Moreover, the insulin injection with insulin shots inevitably restricts a patient’s life style, for example, the hour of rising and meals.

Meanwhile, in case of insulin pumps, an accompanying insulin cannula is put into the human body continuously supplying a little amount of the *short-acting* insulin even in bedtime. Although the duration of the effect of insulin is very low, the short-acting insulin acts faster than the slow-acting insulin. Therefore, a little amount of a continuous dose of insulin can make predictions of blood glucose concentration comparatively easy and its variance is not more than 3% [31].

**Figure 4:**
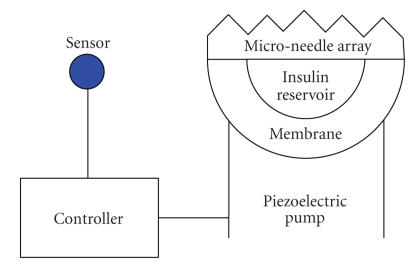
Piezoelectric pump and micro-needle device.

Currently, one of the most promising pump technologies is a piezoelectric fluid device [29]. Piezoelectric pumps operate by applying voltage to a thin lead zirconate titanate (PZT) film. This distorts the film, causing it to pump fluid through an adjoined silicon nitride membrane, shown in Figure 4.

The displacement volume of an unloaded pump can be described as

(1) $$\begin{array}{ll} \Delta V & =\frac{3r^{4}(5+2\mu )(1-\mu )d_{13}U}{4h^{2}(3+2\mu )}, \end{array}$$

(2) $$\begin{array}{ll} K & =\frac{3r^{4}(5+2\mu )(1-\mu )d_{13}}{4h^{2}(3+2\mu )}, \end{array}$$

(3) Δ*V* = *K*Δ*U*,

(4) *Q* = *Kf*Δ*U*,

where *r* is the radius of the membrane in *μ*m, *h* is the thickness of the membrane in *μ*m, *μ* is the Poisson ratio, Δ is the peak-to-peak voltage applied to the pump every period, *d*_13_ is the piezoelectric coefficient, and *f* is the frequency at which voltage is applied to the pump. *K* represents the pump coefficient described by (1). *Q* is the flow rate of the pump.

Note that Δ is linearly proportional to Δ. Likewise, *Q* is also linear for a fixed *f* or a fixed Δ. This permits very simple and intuitive control of the pump by the controller, allowing it to control the flow rate through a variable amplitude signal or a variable frequency signal, depending upon system requirements and the resources available.

Pump reliability is an incredibly crucial factor when considering a blood glucose management system. Pump, sensor, or control failure can lead to incorrect dosages or even a failure to inject. Due to its mechanical nature, pump failure is often the most likely cause of malfunction. This can results from o-ring leaks, air bubbles, bleeding, infection, and clogs. These conditions can easily result in hyperglycemia or hypoglycemia and must be monitored for.

We compare five types of popular insulin pumps in Table 1.

### 5.2. Blood glucose sensor

**Figure 5:**
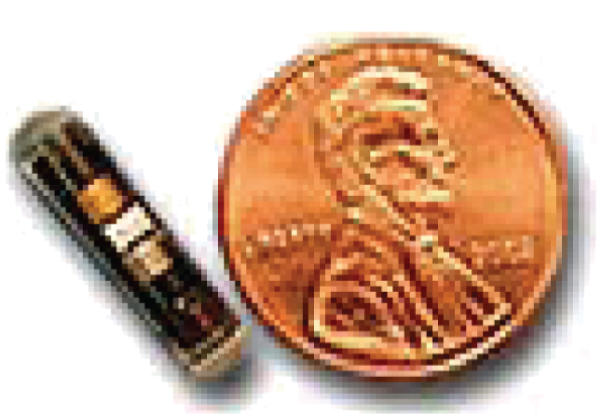
An image shows a miniature size of in vivo blood glucose sensor that is being developed [15]. It is currently proven by FDA that in vivo blood glucose sensors are only safe and reliable in a short period of time (e.g., three days). Thus after the period, they are required to be replaced by a new one regularly to keep their accuracy.

In consideration of easy predictions of blood glucose profile, recently, utilization of both an insulin pump and a blood glucose sensor is very important for the intensive insulin-dependent diabetes therapy. In general, when the system has a capability of the continuous glucose measurement, and with this measurement, a rate of exogenous insulin dosage is automatically or manually adjusted, and the system is called a closed-loop control system. In this system, a glucose sensor may be implanted in the human body as shown in Figure 5 [15]. On the other hand, when the system still counts on periodical glucose measurements, the system is called a partially closed-loop control system. Generally, this latter system utilizes very complex nonlinear calculations or mathematical modeling to pursue its accuracy from few glucose measurements. Besides, insulin dosage plan is designed by a physician with an expert system that performs comparisons between the generated predictions and model cases.

#### 5.2.1. Fingersticks

There are many kinds of blood glucose sensors to be exploited. One of them, fingerstick meter readings, measures blood glucose profiles directly from blood samples taken out of the tip of a finger. This is invasive and can be painful. Additionally, measurements of this sort are only taken a few times per day due to the conscious effort required to perform them [34].

**Figure 6:**
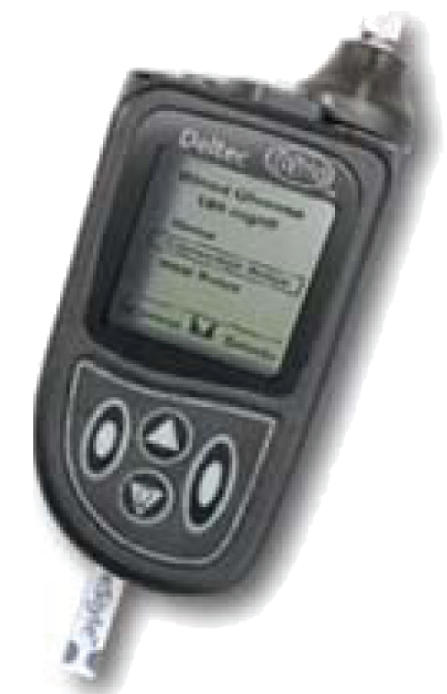
An image shows a fingerstick meter reading. In the use of the sensor, first off, cut the tip of a finger or earlobe bleeding. Then put the blood on a blood glucose sensor, which is a protuberance at the bottom of the module. The result is shown on the display in the middle-top of the module.

In testing a patient blood sample, for example [35], some products require only 0.3 micro liters of the blood sample, but required samplings are at least 4 times per day [36]. Besides, this kind of sensor is also integrated into an insulin pump enhancing portability of the system as well as eliminating a requirement of the manual input of data into the insulin pump, shown in Figure 6 [35]. A drawback of the sensor is that since it invasively samples blood from a finger or earlobe, it is almost impossible to take samples continuously in order to enhance the measurements. Therefore, increasingly, different types of sensors are being developed and used, and they provide a wealth of benefits compared to finger sticks.

#### 5.2.2. Implantable sensors

In addition to the fingerstick meter readings, implantable glucose sensors are available with some limitations. One type of these implantable sensors is needle-type sensors [24]. These needle-type sensors are intravenously or subcutaneously inserted into the body. Intravenous systems monitor blood glucose levels by drawing blood through a vascularly embedded needle [15]. This has the advantage of up to date, real-time BG levels, but at the expensive of being invasive and painful. Additionally, there are long term effects associated with prolonged vascular invasion, making it a suboptimal solution for continuous long-term home monitoring.

Subcutaneous glucose sensors are small electrode devices that can be inserted into the skin in the fatty tissues. This includes collecting a blood sample from the dermis layer of the skin [24], which is located about a tenth of a millimeter into the surface of the body [2]. When the sensors are placed correctly, current proportional to the blood glucose level can be detected and measured. For example, a probe of a subcutaneous sensor is made of an amperometric cell covered by a thin membrane. With this sensor, only needle part is inserted subcutaneously into the body while a processor module stays outside. Due to its shallow position, subcutaneous monitoring can be significantly less painful than finger sticking. The greatest boon of subcutaneous monitoring, however, is that it can be performed continuously in a wearable fashion. This quality enables new types of control techniques to be exploited. The finer the measuring time increment is, the more accurate control methods will be. Continuously glucose monitoring permits real-time signal filtering in attempts to closely regulate changes in glucose due to various factors such as meals, exercise, or sleep patterns.

There are several methods for administering subcutaneous monitoring, including dialysis and open-flow microperfusion. Currently, the most advanced method of subcutaneous monitoring is considered to be microperfusion [24]. Microperfusion entails diffusing interstitial fluids from the dermis into a double lumen catheter where it can be monitored by an external sensor without actually drawing blood.

Also, another type of in vivo sensor is a small piece of module which is completely implanted into a segment of the body of the diabetic. Since these glucose sensors are implanted into the human body, continuous measurement of blood glucose levels will be possible. The authors in [26] demonstrated that samplings were conducted every 5 minutes. The sampled blood glucose profiles are wirelessly transmitted to external receivers that could be a PDA or some other small devices looking like a list-watch. According to the transferred vital data, it is preferable to automatically reconfigure a current insulin delivery rate, but some systems still require the users to manually reconfigure the delivery rate. Although, if realized, the continuously blood glucose monitoring must largely benefit for the insulin therapy, there are many difficulties to implant blood sensors subcutaneously in a long period of time because of physiological reactions that deteriorate the sensors and limit them working sufficiently up to 3 days [21, 24, 37]. For example, MiniMed CGSM System Gold is currently approved by U.S. Food and Drug Administration (FDA) for 3-day use [24, 26].

#### 5.2.3. GlucoWatch

Furthermore, noninvasive blood glucose sensors also exist. Dielectric spectroscopy (DS) is a noninvasive, extracorporeal approach to continuous blood glucose monitoring [2, 24]. DS involves analyzing the electrolyte balance across cells, and comparing those results to known behaviors for differing BG concentrations. One implementation of DS blood glucose monitoring entails coupling an open resonant circuit to the skin. This acts as an RCL sensor (*R* refers to a general organic molecule and CL refers to chlorine), comparing measurement results to derived system models in order to deduct the BG level of the patient.

GlucoWatch is a new glucose monitor from Cygnus Corp., Ill, USA. The monitor straps to the wrist of the patient and uses a patented electrochemical sensor to measure glucose levels in the patient. The GlucoWatch works both continuously and noninvasively, permitting closed loop blood glucose level control. GlucoWatch displays the most recent blood glucose levels of the patient, updating every 20 minutes, and will sound an alarm if the blood sugar level goes above or below predetermined thresholds. The device stores the previous 4,000 readings that can be offloaded for use by a physician. This permits accurate monitoring of long-term insulin dosage regiments and their results.

The GlucoWatch has also recently won FDA approval and is offered for sale in the United States and the UK. The device is actually based upon technology that is almost 100 years old. It takes advantage of the observation that an electric current can selectively transport chemicals through human skin. This transport phenomenon, called *iontophoresis*, has historically been seen as a one-way street, a way to get chemicals into the body. Cygnus scientists and engineers saw an untapped opportunity, creating a device that reverses iontophoresis to get the glucose out. “*A lot of substances can be measured through reverse iontophoresis, but we felt there was a great unmet need for glucose monitoring*,” says Dr. Russell Potts, a biochemist and Cygnus vice president of research [38].

The watch applies a biosensor, called the Autosensor, against the skin that measures BG levels, producing a current proportional to the BG level. A 20-minute analysis cycle starts as the sensor silver-silver chloride iontophoresis electrode applies a 300-microamp current to the skin. For the next three minutes, positive and negative ions travel through the patient skin to the GlucoWatch side-by-side collection discs, which serve as an anode and cathode during glucose extraction. This ion migration acts as a glucose transport, depositing it at the cathode to be measured. The onboard microcontroller then interprets the glucose level into a standard unit of mm/dl.

The DirecNet group conducted a 6-month randomized trial [4] to measure the effects of the GlucoWatch continuous sensor on blood glucose control, hypoglycemia, and quality of life as compared to standard care. At the end of six months, there was no measurable difference in blood glucose control between the experimental and control groups, as measured by A_1_C and mean glucose using the Medtronic retrospective CGMS device. The results also showed that the use of the device had no positive or negative psychological impact on the subjects in the experimental group. These results were puzzling until the usage data was reviewed, which tracked the number of times per week subjects actually used the device. During the first month, 64% of subjects used the device at least twice per week (2.1 ± 0.8). However, by the third month, average use was only 1.6 ± 0.7 times per week, and 7 of the 99 subjects had discontinued use altogether. By the sixth month, the average use was 1.5 ± 0.6 times per week, and 26 of the original 99 subjects had discontinued use. In summary, differences in clinical outcome failed to materialize because an increasing number of subjects stopped using the device. Data gathered from the questionnaires revealed that families felt the information gained from the device was not worth the discomfort and adhesive problems encountered with its use.

**Figure 7:**
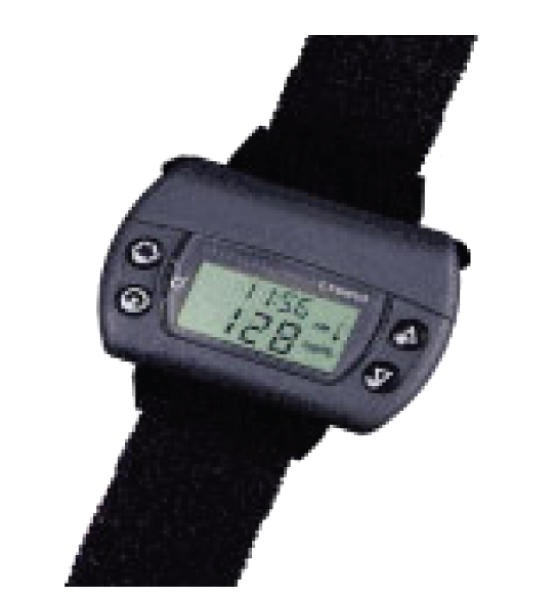
An image shows a list-watch type noninvasive blood glucose measurement [1]. The sensor can avoid damaging the tip of a finger or earlobe to sample blood in a noninvasive way. Therefore, it basically utilizes a quite low electric current to draw blood glucose penetrable through the skin. This sampled glucose is aggregated into a sensing part of the device integrating an electrode that is used for measuring blood glucose levels.

The current version of GlucoWatch, called GlucoWatch G2 Biographer, is shown in Figure 7 [1]. As already mentioned, a list-watch shaped blood glucose sensor utilizes a quite low electric current to draw blood glucose penetrable through the skin [1]. This collected glucose is aggregated into a sensing part of the device integrating an electrode that is used for measuring blood glucose levels. Every measurement is saved in memory, and from efficiently sampled data, the blood glucose level transitions and patterns can be computed. In case of GlucoWatch G2 Biographer, a blood glucose reading period is adjustable in the range of 10 minutes to 13 hours [1]. Although the GlucoWatch already won FDA approval, the noninvasive blood glucose sensors are still supplemental use only rather than used as the main measurements, such as fingerstick blood glucose sensors [1]. Therefore, in case intensive measurements that pursue more accurate vital data are required, noninvasive blood glucose sensors are used supplemental to the conventional testing results. From the results, the best decisions are made by a physician.

#### 5.2.4. Commercial sensors

Here we use a table as follows to compare between four typical commercial glucose sensors (Abbott [39], MiniMed Paradigm [40], MiniMed Guardian, [41] and DexCom [42]) from the following aspects: (1) accuracy of measurements, (2) start-up time, (3) sensor lifetime (with batteries), (4) how they calibrate values, (5) how frequently they display the data, (6) memory size to store the data, (7) transmission distance (from the sensor to a monitor), (8) batteries, (9) monitor size, and (10) alarm system.

### 5.3. Subcutaneous devices

For the sake of the safety, currently, most of the continuous blood glucose sensors being developed are designed to be put into subcutaneous tissues. Several methods for subcutaneous injection have been proposed and implemented in order to take advantage of the benefits of subcutaneous injection [15]. Manual injection systems typically include a subcutaneous needle or injection pen, which are applied according to a physician prescribed schedule. Continuous control systems generally deal with its time-to-act shortfalls through appropriate algorithms and choice of insulin.

#### 5.3.1. Open-flow microperfusion (OFM)

In the open-flow microperfusion (OFM), in order to capture the blood glucose levels subcutaneously, a needle-like double-lumen catheter is inserted into the adipose tissue [24]. Firstly, it is supported by a steel mandril, but eventually this steel mandril is switched by the inner cannula. In the double-lumen catheter, a perfusion fluid is filled in the annular space between the inner cannula and the outer catheter. Because of partial equilibration between the interstitial fluid and the perfusate, aspiration occurs. Thus the glucose sensor outside the body can capture glucose from the aspirated fluid.

In this method, since the implantable part of sensor is just like a needle for the subcutaneous insulin injection, it is comparatively easy for patients to insert the sensor.

**Figure 8:**
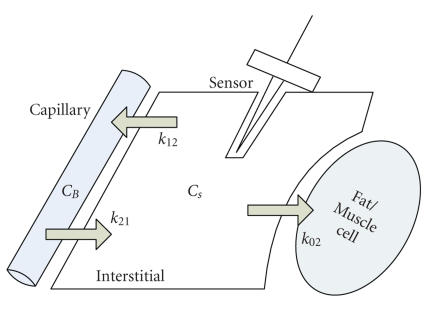
A capillary and interstitial compartmental model [43, 44]. glucose uptake, and diffusions between capillary and interstitial tissues are represented as arrows.

Compared to actual clinical practices, where blood samples are picked from intravenous injections directly measuring current blood glucose profiles, the subcutaneous monitoring has different results from those from the capillary monitoring due to the first-order lag [43]. Therefore, in the subcutaneous monitoring, it is required to approximate actual blood glucose profiles from subcutaneously captured data by finding a relation between blood and subcutaneous glucose profiles. A capillary and interstitial compartment model is shown in Figure 8.

To find a mathematical relation between the blood and subcutaneous glucose profiles, [43] utilizes a first-order lag to describe the subcutaneous glucose dynamics in terms of the blood *C_B_* and subcutaneous glucose profiles *C_S_* as follows:

(5) $$\frac{dC_{s}}{dt}=-(k_{02}+k_{12})C_{s}+k_{21}\frac{V_{1}}{V_{2}}C_{B},$$

where *k*_02_ is an uptake rate of glucose in the subcutaneous tissues, *k*_12_ and *k*_21_ are diffusion rates between the blood and subcutaneous compartments, and *V*_1_ and *V*_2_ represent the volumes of both blood and subcutaneous tissues, respectively.

The same first-order model for the subcutaneous glucose dynamics model is derived in the paper [45], which showed that an actual value of *k*_12_ is within the range of 0.04 to 0.11 min^−1^. Also, [45] figured out from rat studies that a value of *k*_02_ is 0. Moreover, a first-order model can be written as

(6) $$\frac{dy}{dt}=ay+bu=-\frac{1}{\tau }y+\frac{k}{\tau }u,$$

where *u* and *y* are the input and output perturbations, respectively, and *k* and **τ** are the gain and time constants. Thus comparing this standard form to an equation derived by [43, 45], *k* and **τ** are solved as follows:

(7) $$k=\frac{k_{12}}{k_{02}+k_{12}},\;\;\tau =\frac{1}{k_{02}+k_{12}}.$$

In [43], by setting *k* and **τ** to 1 and 12 minutes, respectively, a simple simulation model is generated with the reduction of blood glucose from 200 to 100 mg/dl and a first-order decay time is 75 minutes. Subcutaneous measurement has a noise with a standard deviation of 1 mg/dl.

#### 5.3.2. Microneedle array

One subcutaneous device to be proposed is microneedles made out of silicon [29]. Microneedles for insulin injection are designed to penetrate through several layers of skin: the stratus corneum layer, the epidermis layer, and part of the dermis layer. This method of injection punctures through far fewer nerve cells than classic intravenous injection, while still having access to the dermis layer, which is rich in blood vessels. This results in a far less invasive and painful injection experience for the patient.

**Figure 9:**
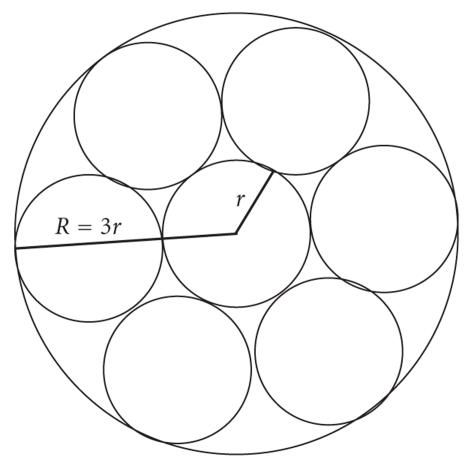
Microneedle honeycomb formation.

An expansion upon this idea has been to fabricate an array of microneedles. This provides many benefits, with few disadvantages. The more microneedles used, the smaller in diameter each needle needs to be in order to facilitate a load-free flow of insulin into the body. Shrinking the size of the needles translates into an even less painful experience, while maintaining the same operational insulin flow rate. Additionally, an array of needles provides redundancy to the injection system, safeguarding against reduced flow due to channel blocking or clotting. Flow rate, *Q*, can be found as follows, given *n* needles, pressure change Δ*P*, needle length *L*, needle radius *r*, and fluid viscosity *μ*, where the relationship of *R* and *r* can be found in Figure 9:

(8) $$\begin{array}{ll} R & =\frac{8\,\;\mu \text{L}}{\pi r^{4}},\\ Q & =n\frac{\Delta P}{R},\\ Q & =n\Delta P\left(\frac{\pi r^{4}}{8\,\;\mu \text{L}}\right). \end{array}$$

Note that the flow rate is linearly proportional to the number of microneedles, given a fixed radius. Unfortunately, the flow also decreases by an order of 4 as radius is reduced.

Replacing a single large needle with a honeycomb formation of 7 tightly packed microneedles (each one-third of the diameter of the original needle) produces a flow of only 8.6% of the original flow for the same surface area. This observation makes evident the fact that due to the high-order impact of needle radius, the skin area required for a microneedle array increases exponentially as the size of the needles are reduced. In practice, the flow rate required is actually quite low, however, making this exponentially increasing contact area functionally negligible to the end user, due to its still relatively small size. As far as the patient is concerned, the increase in skin surface required is well worth the significantly reduced invasiveness and pain of the device.

## 6. CONTROLLING BLOOD GLUCOSE PROFILES

By deploying devices described in the previous section as well as understanding the mechanism of the insulin-glucose dynamics, blood glucose profiles can be controlled systematically. However, in general, not only the insulin-glucose dynamics but also any other human metabolism are so complicated that they can be hardly quantified. Therefore, arguably, the most complex component of blood glucose management is the control domain. There are several classes of solutions to this problem, ranging in complexity, prerequisite knowledge, and feedback.

To do this, researchers first empirically or with the fundamental methods derive a couple of mathematical equations about the insulin-glucose dynamics [46, 47]. While the empirical methods largely depend on the input-output data from experiments, the fundamental methods basically derive them from the knowledge about the human internal functions which are already familiar enough. After constructing a mathematical model of the insulin-glucose dynamics, an insulin-dependent diabetes therapy is designed. The goal of the therapy is to mitigate the excessive glucose production by artificially secreting exogenous insulin systematically. The insulin secretion must follow the actual metabolic functionality.

The control methods are broadly categorized in three categories: open-loop control, closed-loop control, and partial closed-loop control methods [15]. This section briefly explains these three control methods with applications. More detailed explanations can be found in our survey on control methods in [5].

### 6.1. Open-loop control

Open-loop control methods are occasionally called the *programmed* insulin infusion system. One type of this method is one that was developed by Case Western Reserve University, and this system is considered to be one of the most intelligent programmed insulin infusion products that deal with the noninsulin-dependent diabetics. The idea is that from an analysis of the insulin curve in the nondiabetic, it was turned out that the curve approximately traces a combination of a double exponential curve and a basal insulin infusion [48]. According to this mathematical model, an intravenous insulin delivery system was designed such that it followed the real pancreas functionality of the nondiabetic. The system utilized a portable cart containing the control system, the insulin pump, power supplies, and insulin reservoir so that the patient could move around with the devices. The insulin pump delivers low-concentration insulin and updates the insulin delivery rate every 30 seconds. Because of its simplicity, the system can be set up and operated by nurses [48].

### 6.2. Closed-loop control

**Figure 10:**
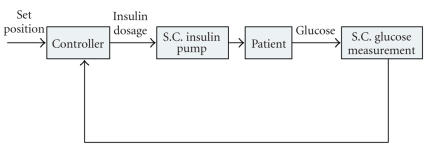
A control flow of closed-loop control models [15]. Entire loop of the control is closed from outside by utilizing an insulin pump and in vivo blood glucose sensors. Both insulin injections and glucose measurements are carried out subcutaneously.

At first, a flow of the closed-loop control scheme is shown in Figure 10. The closed-loop system completes its operating cycle within the system and no external interaction to diabetic patients is required [15, 24]. In other words, the closed-loop control uses the feedback from the output. Typically, the closed-loop system for type 1 diabetes therapy utilizes the glucose sensor and schematically consists of three phases: blood glucose measurements, insulin demand calculation, and insulin injection. The closed-loop system repeats this sequence.

Several papers already proposed applications and mathematical models to suffice this model. Such applications include the pole-assignment strategy, self-tuning adaptive control, model predictive control, and neural predictive control.

In short, the pole-assignment strategy calculates the insulin injection rate (IIR) according to the current blood glucose levels plus trends of the blood glucose levels as well as the pharmacokinetics of insulin infusion. Although the calculation is very simple, the pole-assignment strategy is time consuming because of repeatedly evaluating the model parameters only from the current measurements. On the other hand, the self-tuning adaptive control utilizes a recursive computation of the model parameters to generate the IIR faster than the pole-assignment strategy [15].

### 6.3. Partially closed-loop control

**Figure 11:**
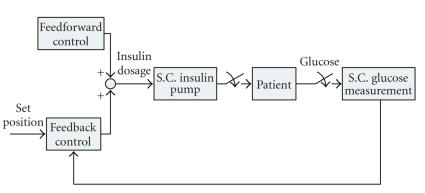
A control flow of partially closed-loop insulin therapy models from [15]. In general, in this case, insulin dosages are supported by an expert system that generates an optimal insulin dosage from a comparison between a model case and a prediction of a future blood glucose transition.

A flow of the partially closed-loop control is shown in Figure 11. Like the closed-loop control models, the partially closed-loop control utilizes the external devices, such as insulin pumps and blood glucose sensors. The major difference between the closed-loop and partial closed-loop control methods is that the closed-loop control only depends on the feedback from the output, while the partial closed-loop relies on the physician assessment of the condition as well.

Thus in a partially closed-loop control of the insulin dependent diabetes therapy, measurements are conducted three to seven times per day, and insulin injections are also performed three to four times under the supervision of a physician. These decisions, for example, the number and type of insulin injections and insulin dosage [15], are made according to model-based or algorithmic-based decision support systems, such as DIAS, AIDA, and T-IDDM [15]. Insulin injections are usually performed by using the subcutaneous (SC) route due to its management and safety.

For example, unlike the pole-assignment strategy or the self-tuning adaptive control in the closed-loop control scheme, AIDA simulates the effect of the exogenous insulin injections and virtually models insulin-glucose dynamics based on their physiological rules around metabolism of a single glucose compartment rather than determining the IIR [15, 43, 44]. According to the virtually modeled insulin-glucose dynamics, a physician determines the appropriate exogenous insulin injection. Likewise, DIAS is a nonlinear model of the blood glucose-insulin system based upon real-life parameters versus, simply, the blood glucose measurements [49]. It incorporates qualitative and quantitative inputs from the user, including the blood glucose levels, meals, and past insulin injections.

In addition to DIAS, AIDA, and T-IDDM, the Bergman model and physician prescribed regiment are also categorized in the partially closed-loop control models [5].

## 7. CONCLUSION

It is evident that reliable solutions to diabetes management are highly sought after and researched. The health benefits associated with intensive diabetes treatment can save untold lives and make life far more comfortable for many others. Additionally, with the number of diabetes sufferers increasing annually, and the disease already costing well over *$*100 million per year in health care expenses, it is a very lucrative market. Effective solutions can help mitigate and control the effects and tolls of diabetes in terms of both health and economic impact.

Personal *artificial pancreas* devices are coming into greater use, spurred on by improvements in technology and an increasing demand. Appropriate technologies must be developed and exploited in order to improve upon today’s methods. Increased miniaturization of sensors, pump, and controls continuously improve the portability of personal continuous control devices, thereby increasing their ubiquity among insulin-dependent diabetes patients.

This paper summarizes diabetes and its therapies. Currently, diabetes is considered not to be cured by any medical treatment. However, since the Diabetes Control and Complication Trial demonstrated that, by well-controlling, the glucose metabolism for the diabetic, especially type 1 diabetes patients, expected emergence of complications can be delayed or protected, several techniques to replace exogenous insulin to the lack of endogenous insulin in order to suppress the rise of the amount of glucose in blood are developed, that is, the insulin-dependent diabetes therapy.

Besides, to support the insulin therapy, several medical devices concerning diabetes are being developed. Examples of these devices are insulin pumps and blood glucose sensors. In this paper, a couple of examples of insulin pumps and blood glucose sensors are presented. Especially, in blood glucose sensors, three types of them are briefed: the fingerstick meter reading, in vivo, and the noninvasive blood glucose measurement, and each of them has its advantages and disadvantages. For example, although, if they are available, in a long time period, the in vivo blood glucose sensors largely benefit for modeling the blood glucose transitions and consequently benefit for the insulin therapy, they are only approved for a short time use currently, for example, only a 3-day use is approved by U.S. Food and Drug Administration (FDA). The insulin therapy using the in vivo sensors and insulin pumps are usually called the closed-loop control.

In conclusion, in the near future, several research intentions will be considered. The first is the early development of long-term available in vivo blood glucose sensors. There is no explanation about this direction because it is believed that the emergence of long-term in vivo sensors should enhance the blood glucose measurement and consequently ease the predictions and plans for the insulin therapies to avoid both hyper- and hypoglycemia. The second is the development of a more accurate but simpler mathematical model for the insulin-glucose dynamics. This direction will also enhance the insulin therapy largely in the diabetic treatment by partially closed-loop control. For more details about the control theory in diabetes, please refer to our survey paper in control methods [5]. In addition to these two directions, the paper [15] points out two more directions: one is the development of decision support systems, and the other is the development of the noninvasive blood glucose measurement.

## Figures and Tables

**Table 1:** Specification comparison of commercial insulin pumps.

Company	Animas	Deltec	Disetronic	MiniMed	Insulet
Model	IR-1250 [11]	Cozmo [12]	Spirit [13]	Paradigm* *522/722 [14]	OmniPod [33]

Dimensions	79 × 51 × 19	80 × 47 × 24	80 × 56 × 20	522: 51 × 79 × 20	Pod: 41 × 61 × 18
				722: 51 × 79 × 20	Pda: 66 × 110 × 26

Screen size	992 sq mm	870 sq mm	Unavailable	774 sq mm	1,848 sq mm on PDA controller

Basal delivery	Every 3 minutes	Every 3 minutes	Every 3 minutes	Varies	Information unavailable

Basal temperature	Initially −90% to +200%, varies for every half an hour	Varies from 0% to 200% with an increment of 5% for every half an hour	Varies from 0% to 200% with an increment of 10% for every half an hour	+/−0.1 increment as single basal rate for 0.5 to 24 hours	Information unavailable

Carb and correction factors	Manual entry and assist from EZ manager	Manual carbohydrate, BG from attached CoZ monitor	Manual carbohydrate, BG from Accu-check BG monitor	Manual carbohydrate, BG from BD meter or manual entry	Information unavailable

Battery	AA lithium × 1	AAA × 1	AA × 1 Alkaline or Rechargeable	AAA	AAA × 2

Motor	DC	DC	DC	DC	Stepper

Memory	Nonvolatile: 600 bolus, 270 basal, 120 daily totals, 30 alarms, and 60 primes	Nonvolatile: 90 days of basals, carbohydrates, boluses, correction boluses, and alarms	Nonvolatile: 90 days history recall of last 30 boluses, alerts, daily insulin totals, and temporary basal rate increase	4000 events volatile: 24 boluses, and 7 days totals	90 days of data

Extra features	Clip-on covers, personalized carbohydrate, and correction factors, tracks residual bolus insulin	Carbohydrate and correction factors, tracks residual bolus insulin, detailed records of pump, and daily bolus total correction	Availability of different types of user menus, icon and menu driven programming, backlight display, and reversible display screen	Extended bolus, auto off	Integrated free style meter, 1000 common foods in PDA

**Table 2:** Specification comparison of commercial blood glucose sensors.

Features	Abbott freestyle navigator [39]	MiniMed paradigm real-time system [40]	MiniMed guardian real-time system [41]	DexCom [42]
Accuracy	Varies	Consensus error grid: 98.9%* * *A* mard(mean) −19.7% (median) −15.5%	Consensus error grid: 98.9%* * *A* mard(mean) −19.7% (median) −15.5%	Consensus error grid: 95.4%* * *A* mard(mean) −49% (median) −15.9%

Startup initiation time	10 hours	2 hours	2 hours	2 hours

Sensor life	5-day wear indication	Above 72 hours	Above 72 hours	Above 72 hours

Calibration method	Requires calibration at 10, 12, 24, and 72 hours after the insertion of the sensor	Alarms when calibration value is not entered on time. First and second calibration should be done for 2 and 6 hours after insertion	Alarms when calibration value is not entered on time. First and second calibration should be done for 2 and 6 hours after insertion	First calibration after 30 minutes and then for every 12 hours. Manual calibration is not possible

Frequency of display	Every 1 minute	Every 5 minutes	Every 5 minutes	Every 5 minutes

Transmitter memory	—	Yes, the transmitter stores missed data for up to 40 minutes	Yes, the transmitter stores missed data for up to 40 minutes	No, transmission lost is data lost

Range of monitor to transmitter	10 feet	6 feet	6 feet	5 feet

Monitor batteries	Uses 2 AAA batteries with replacement for every three months	No separate monitor required. Uses insulin pump	Uses 2 AAA Batteries. Indication is set for chance of battery	Uses rechargeable batteries

Monitor size	3“ × 2.5”	Separate monitor is not available. Uses insulin pump for display	3“ × 2.7”	3“ × 2.5”

Alarms on user-set low and high thresholds	Applicable	Applicable	Applicable	Applicable
